# Draft Genome Sequence of *Streptococcus anginosus* BVI, a New Vaginal Pathogen Candidate

**DOI:** 10.1128/genomeA.01417-16

**Published:** 2016-12-15

**Authors:** Andres Zuñiga-Bahamon, Fabian Tobar-Tosse, Jose Guillermo-Ortega, Daniel Wibberg, Andreas Tauch

**Affiliations:** a Department of Basic Sciences for Health, Facultad de Ciencias de la Salud, Pontificia Universidad Javeriana Cali, Cali, Colombia; b Center for Biotechnology (CeBiTec), Bielefeld University, Bielefeld, Germany

## Abstract

*Streptococcus anginosus* is a pathogen implicated in urogenital and gastroinstestinal tract infections. Here, we report the draft genome sequence of *S. anginosus* BVI, isolated from a bacterial vaginosis patient attending a prenatal care unit in Cali, Colombia. The genome sequence of BVI consists of 2,014,025 bp, encoding 2,008 predicted proteins.

## GENOME ANNOUNCEMENT

*Streptococcus anginosus* and related streptococci have been primarily recognized as commensals of the human mucosa (1), since the full appreciation of their clinical significance was hampered for a long time due to difficulties in correct species identification (2, 3). In recent years, members of the *S. anginosus* group have been detected as potential pathogens in abscesses and blood cultures, and they also play a role in cystic fibrosis (1, 4). In other studies, *S. anginosus* has been recognized as an associated pathogen of different body sites, such as the oral cavity and the urogenital and gastroinstestinal tracts (5, 6). Although *S. anginosus* has been found in the human vaginal and urinary microbiome (7–10), a genome of an *S. anginosus* isolate found in the vaginal canal has not been sequenced so far.

Here, we report the draft genome sequence of *S. anginosus* BVI that was isolated from the vaginal fluid of a pregnant patient with bacterial vaginosis attending routine prenatal care. The respective specimen was collected with a sterile swab and cultured on human blood Tween (HBT) agar in 5% CO_2_. Colonies were subcultured on HBT agar, and genomic DNA was purified according to a salt-based miniscale protocol (11). The genomic DNA was sequenced by the paired-end technique on the MiSeq system (Illumina). The sequencing run yielded 699,061 reads accounting for ~203 Mb of total DNA information. In this way, an approximately 100-fold coverage was achieved for the 2.0-Mbp genome of *S. anginosus* BVI. The assembly was carried out with the GS *De Novo* Assembler software (version 2.8). The draft genome sequence consists of 26 contigs with a total size of 2,014,025 bp and a G+C content of 38.9%. Pairwise DNA comparisons with the complete genome sequences of *S. anginosus* strains C238 (accession no. CP003861), SA1 (accession no. CP007573), and J4211 (accession no. CP012805) by average nucleotide identity (ANI) analysis (12) revealed values above 95% for each genome pair, confirming the taxonomic classification of the BVI isolate as *S. anginosus*.

The annotation of the draft genome sequence was performed with the Prokka version 1.11 software (13) and the GenDB 2.4 platform (14)). The draft genome sequence of *S. anginosus* BVI contains 2,008 protein-coding regions and 46 tRNA genes. As *S. anginosus* BVI was isolated from a patient diagnosed with bacterial vaginosis, the exploration of virulence factors is of importance to understand bacterium-host interactions and potential virulence mechanisms. Analysis of the genome data by using the virulence factor database VFDB (15) presented a significant load of iron uptake systems classified as nonspecific virulence factors and of secretion systems classifed as offensive virulence factors. *S. anginosus* BVI probably represents a new vaginal pathogen that can be isolated from cases of bacterial vaginosis.

### Accession number(s).

The *S. anginosus* BVI whole-genome shotgun project has been deposited in ENA under the accession no. FMKB00000000. The version described in this paper is the first version, FMKB01000000.
