# Correction for Zuñiga-Bahamon et al., Draft Genome Sequence of *Streptococcus anginosus* BVI, a New Vaginal Pathogen Candidate

**DOI:** 10.1128/genomeA.00112-17

**Published:** 2017-03-09

**Authors:** Andres Zuñiga-Bahamon, Fabian Tobar-Tosse, Jose Guillermo-Ortega, Daniel Wibberg, Andreas Tauch

**Affiliations:** a Department of Basic Sciences for Health, Facultad de Ciencias de la Salud, Pontificia Universidad Javeriana Cali, Cali, Colombia; b Center for Biotechnology (CeBiTec), Bielefeld University, Bielefeld, Germany; c Programa de Doctorado en Ciencias Biomédicas, Universidad del Valle, Cali, Colombia

## AUTHOR CORRECTION

Volume 4, no. 6, e01417-16, 2016, https://doi.org/10.1128/genomeA.01417-16. Page 1: The affiliation footnotes to the byline and the affiliation line should read as given above.

Page 1: The Acknowledgments should include the following. “DNA extraction and complementary molecular laboratory equipment was provided by Programa de Doctorado en Ciencias Biomédicas, Universidad del Valle, Cali, Colombia.”

Page 1: The Funding Information should include the following. “This work, including the efforts of Andres Zuñiga-Bahamon, was funded by Programa de Doctorado en Ciencias Biomédicas, Universidad del Valle, Cali, Colombia.”

